# Methyl jasmonate treatment alleviates chilling injury and improves antioxidant system of okra pod during cold storage

**DOI:** 10.1002/fsn3.3241

**Published:** 2023-02-01

**Authors:** Yunfen Liu, Yu Liu, Qiumei Chen, Feilong Yin, Mubo Song, Wen Cai, Liang Shuai

**Affiliations:** ^1^ College of Food and Biological Engineering Hezhou University Hezhou Guangxi China; ^2^ Guangxi Key Laboratory of Health Care Food Science and Technology Hezhou University Hezhou Guangxi China

**Keywords:** antioxidant system, chilling injury, factor analysis, methyl jasmonate, okra pod

## Abstract

Okra pod is sensitive to low temperature, which results in chilling injury under improper low‐temperature storage. This study aimed to evaluate the effect of different concentrations of methyl jasmonate (MeJA) treatment on okra pod stored at 4 ± 1°C for 12 days and illuminate the mechanism of MeJA alleviating chilling injury. Compared to the control, MeJA treatments maintained lower relative electric conductivity (REC), chilling injury (CI) degree, and lignin content, as well as higher total soluble solids, total soluble sugar, pectin content, and chlorophyll content. The factor analysis was applied to comprehensively evaluate the effects of MeJA so that 1 μmol/L MeJA was screened as the optimum concentration to maintain the okra quality throughout the storage time. In contrast with control, MeJA not only accelerated the generation of antioxidant substances (phenolics and flavonoids) but also increased the superoxide dismutase (SOD), catalase (CAT), ascorbate peroxidase (APX), and peroxidase (POD) activity, inhibited malondialdehyde (MDA), hydrogen peroxide (H_2_O_2_) content accumulation, and the polyphenol oxidase (PPO) activity. This work confirmed that MeJA could effectively alleviate chilling injury and maintain the quality during cold‐stored by regulating reactive oxygen species (ROS) metabolism. These results provide theoretical guidance for the application of MeJA in okra storage and preservation.

## Graphical abstract

MeJA treatment has maintained the quality of okra during the cold storage period. The optimal concentration of MeJA was 1 μmol/L which was selected through a comprehensive analysis of quality indicators by factor analysis. MeJA treatment alleviates chilling injury of the okra pod through activating the antioxidant system.

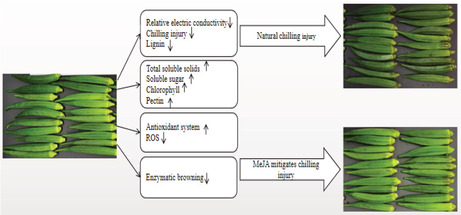

## 1 INTRODUCTION

Okra (*Abelmoschus esculentus* L.) belongs to the Malvaceae family and is a kind of important vegetable crop distributed in tropical and subtropical areas (Patel et al., 2016). It contains rich nutrients like protein, vitamin C, calcium, flavones, pectin, and dietary fiber, and is known as a new health‐protective vegetable in China for its medicinal uses (Kanwal et al., 2020; Phornvillay et al., 2020). It can help with anticancer, antitumor, digestion, confer liver protection, and other health effects (Elkhalifa et al., 2021; Esmaeilzadeh et al., 2020). Okra pods are easily perishable due to their high water content and respiratory rate, resulting in being inedible due to wilting, lignification, color fade, senescence, as well as, sometimes, pathogens during ambient storage (Patel et al., 2016; Sun et al., 2021). Okra pods stored at a low temperature below 10°C could delay senescence, maintain quality, and extend storage time, but is threatened by chilling injury (Finger et al., 2008). The chilling injury symptom on okra are always characterized by pitting, surface browning, and spots (Huang et al., 2012). Generally, some symptoms develop when returning to warmer temperature, which seriously affects the commodity value. Although, a few methods have been reported to reduce chilling injuries of stored okra such as nitric oxide (NO) (Sun et al., 2021), 1‐Methylcyclopropene (1‐MCP) (Huang et al., 2012), putrescine (Phornvillay et al., 2019), Methyl jasmonate (MeJA) (Boonyaritthongchai et al., 2013), and vinyl wrapping (Rekoumi et al., 2012). It is still necessary to find an effective method to reduce chilling injury and illuminate the mechanism.

Previous research showed the chilling injury induced by excessive reactive oxygen species (ROS). Cold stress stimulates the accumulation of ROS, mainly composed of hydrogen peroxide (H_2_O_2_), resulting in lipid peroxidation of the cell membrane and protein degradation (Li, Min, et al., 2021; Li, Yang, et al., 2021; Mohammadi et al., 2021). The antioxidant defense system incorporates antioxidants such as phenolics, ascorbic acid, and flavonoids; antioxidant enzymes such as superoxide dismutase (SOD), catalase (CAT), ascorbate peroxidase (APX), and peroxidase (POD), which are activated to scavenge ROS (Irshad et al., 2021; Sinha et al., 2021), thereby reducing cell membrane damage. In addition, chilling‐induced surface browning results from polyphenol oxidase (PPO) oxidation of phenolics (Liu et al., 2022). Thus, it is essential to improve the antioxidant system to maintain quality and extend the shelf‐life of harvested okra.

Methyl jasmonate (MeJA) is a plant hormone as a signal molecule involved in plant growth and development progress and response to various abiotic and biotic stress (Faizy et al., 2021; Laura et al., 2019; Yu et al., 2019). In recent years, MeJA was applied to improve the chilling resistance of fruits and vegetables such as loquat (Cao et al., 2010), tomato (Li, Yang, et al., 2021), pepper (Seo et al., 2020), and pineapple (Sangprayoon et al., 2019). The reduction of chilling injury by MeJA might be attributed to the enhancement of the antioxidant defense system and lower malondialdehyde (MDA) content, which retains the fruit quality. However, the information on the application of MeJA on okra is scanted, with only one study indicating that MeJA would reduce chilling injury. In this study, the aim was to determine the effects of MeJA on okra exposed to low temperatures and whether alleviating chilling injury related to the antioxidant system.

## 2 MATERIALS AND METHODS

### 2.1 Plant materials and treatments

Fresh okra pods (‘Wufu’) were harvested from the plantation in Hezhou city, Guangxi Province. Fruits without mechanical injury, disease blemished, uniform size, and maturity were selected as experimental materials. The fruits were cleaned with tap water, then air‐dried. Moreover, 720 fruits were divided into four groups randomly. The fruits of four groups were immersed in different concentrations of MeJA (0.1, 1, 10 μmol/L) and distilled water (as CK) for 10 min at 25°C, respectively. Then the fruits were air‐dried. Following treatments, the fruits were placed in transparent PE bags (thickness for 0.06 mm) and then stored at 4 ± 1°C, with a relative humidity of 80–90% for 12 days. Peel samples were taken at 3‐days intervals during storage and then frozen in liquid N_2_ for further analysis. Each experiment sets three replicates.

### 2.2 Measurement of relative electric conductivity

Relative electric conductivity (REC) was determined by the method of Liu et al. (2016) with some modifications. Twenty discs (thick 1–2 mm, diameter 5 mm) of okra peel were taken by steel core borer, rinsed three times with distilled water, and incubated in 25 mL deionized water at 25°C for 2 h. Then the initial electric conductivity was measured which recorded as C_0_, the peel discs were boiled for 15 min and then cooled to 25°C, the total electric conductivity was recorded as C_1_. The relative electric conductivity (%) = (*C*_0_/*C*_1_) × 100.

### 2.3 Chilling injury assay

The degree of chilling injury (CI) was assayed by the extent area of the surface browning or pitting, which was described by Boonyaritthongchai et al. (2013) with small changes. The CI was determined using the following scale: 0, 0% area; 1, ≤5% area; 2, 5%–25% area; 3, 25%–45% area; 4, ≥45% area. The CI index was calculated as: [(NY × Y)]/4∑NY, where Y represents chilling injury severity (0–4), and NY represents the number of okra pods with the corresponding severity score.

### 2.4 Determination of lignin content

Lignin content was determined according to the method by Lei et al. (2012) with slight modification. 10 g of dried samples was boiled with 30 g/L sodium dodecyl sulfate for 1 h, centrifuged at 8000 r/min for 15 min. The residue was washed with acetone, boiled with 2 mol/L HCl for 1 h, cooled to room temperature, and then filtered. The residue was washed with acetone and hydrolyzed with 86% H_2_SO_4_ for 1 h. In addition to four volumes of distilled water were added, the mixture boiled for 1 h, after filtering, the remaining residue was dried at 80°C to constant weight for lignin assay.

### 2.5 Total soluble solids and total soluble sugars content assay

Total soluble solids (TSS) content was determined using a digital refractometer (PR‐32α; Atago Co., Ltd.). 1 g of fresh okra fruits was homogenized with 5 mL distilled water. After centrifugation at 5000 r/min for 10 min, the supernatant was collected. The result was expressed as percent soluble solids.

Total soluble sugars content was assessed by the method of Maina et al. (2017) The results were expressed as OD_540_/g.

### 2.6 Measurement of pectin content

Pectin content was measured following the method by Lei et al. (2012) and Xue et al. (2020), with a small modification. In brief, 1 g of samples were well homogenated with 10 mL 95% ethanol and extracted for 30 min in a boiling water bath, then cooled to room temperature. Centrifugated at 10,000 r/min for 15 min, removed the supernatant. The residue was washed with 95% ethanol three times and air‐dried. Distilled water was added into the residue to hydrolyze and then filtered. The filtrate was used to determine water soluble pectin, and the residue was digested with 0.5 mmol/L H_2_SO_4_ at 100°C for 1 h, then cooled to room temperature, and collected the supernatant for protopectin analysis. Pectin content (%) = water soluble pectin (%) + protopectin (%).

### 2.7 Measurement of chlorophyll content

The chlorophyll content was assayed according to the method described by Zhang et al. (2019) with minor changes. One gram of okra pods was well ground and added with 50 mL precooled 80% acetone, centrifugated at 12 000 rpm for 15 min. The supernatant was collected and used for absorbance measurement at 645 nm and 663 nm. The content was calculated following Arnon's equations (Arnon, 1949).

### 2.8 Measurement of total phenolic and flavonoid content

The determination of total phenolic and flavonoid contents was followed by Pirie and Mullins (1976) with slight modification. 1 g sample powder was homogenated in 8 mL of precooled 1% HCl‐methanol and then centrifuged at 12 000 r/min for 20 min at 4°C. The supernatant was measured at 280 nm and 325 nm, respectively. The total phenolic content was expressed as OD_280_, and the flavonoid content was represented as OD_325_.

### 2.9 Measurement of MDA and H_2_O_2_ content

The MDA content was measured using a thiobarbituric acid reaction, and the method was followed by Zheng et al. (2019) with slight modification. 1 g of okra powder was homogenized with 4 mL precooled 10% trichloroacetic acid (TCA) and then centrifuged at 12 000 r/min for 20 min at 4°C. 1 mL supernatant was blended with 2 mL 0.67% thiobarbituric acid (TBA), reacted at 95°C water bath for 30 min, and cooled immediately in ice. The absorbance was documented at 450 nm, 532 nm, and 600 nm. The content was calculated following the formula as MDA content (μmol/g) = 6.45 × (OD_532_–OD_600_)–0.56 × OD_450_.

The H_2_O_2_ content was assayed according to Liu et al. (2016) with some modifications. 2 g of sample was well ground and homogenized with 6 mL precooled acetone. Following that, centrifuged at 12 000 r/min for 20 min at 4°C, the supernatant was collected. The H_2_O_2_ content was calibrated to a standard curve.

### 2.10 Antioxidant enzymes assay

Extraction of antioxidant enzymes, including SOD, CAT, APX, and POD, was performed according to Zhang et al. (2015) and Sun and Li (2017) with minor modification.

SOD activity was assayed according to the method reported by Pan et al. (2020) The definition of SOD activity is the inhibition of 50% of the nitro blue tetrazolium (NBT) photo oxidation–reduction rate, and the result was expressed as U/g.

CAT activity was determined according to the previously used method (Liu et al., 2016). One unit of CAT activity was defined as the increased absorbance value of 0.01 per minute at a wavelength of 240 nm. The result was expressed as U/min/g.

APX activity was determined by the method described by Zhao et al. (2020) One unit (U) activity was designed as the increase absorbance of the reaction system by 0.01 per minute at a wavelength of 290 nm. Moreover it was expressed as U/min/g.

POD activity was determined using the method reported by Polle et al. (1994) based on the guaiacol oxidation by H_2_O_2_ at 470 nm. One unit (U) activity was defined as the required enzyme of an increase at the absorbance of 0.01. Enzyme activity was expressed as U/min/g.

PPO activity was assayed using the method described by Zheng et al. (2019) with some modifications. 4 mL of the reaction mixture containing 0.1 mL crude enzyme and 3.9 mL 0.1 mol/L pyrocatechol was incubated in a water bath at 35℃ for 30 min. The absorbance was recorded at 480 nm. One unit (U) of PPO activity was defined as U/min/g.

### 2.11 Comprehensive assessment of quality indicators of different treatments on okra fruit

Multivariate analysis and statistical methods have been extensively applied, by replacing most of the initial information with a few extracted factors, as well as factor dimension reduction, for comprehensive analysis of the storage quality of fruits and vegetables (Lei et al., 2018; Xue et al., 2020; Zhu et al., 2016). All variables were standardized by transforming the value into z‐scores. The factor analysis provides a comprehensive overview of the effect of MeJA on okra.

In order to analyze the correlation between the physiological parameters during the storage period, Pearson's correlation coefficients were performed.

### 2.12 Statistical analysis

All data were obtained from three replicates for each treatment and expressed as means±standard error (SE). Factor analysis was performed using SPSS (version 19.0; SPSS Inc.). Significance analysis was conducted with one‐way ANOVA multiple comparison (*p*< .05). The graphics were constructed by Excel and Origin 8.0.

## 3 RESULTS

### 3.1 Effect of MeJA treatment on quality in okra during storage

The quality candidates, including REC, CI degree, lignin content, total soluble solids, soluble sugar, pectin content, and chlorophyll content, were shown in Table 1. The results showed that the REC of 1 and 10 μmol/L MeJA was lower than CK, especially 10 μmol/L, which was significantly lower than CK after 3 days (*p* < .05), the REC was 14.5% lower than CK until the end of storage time. The CI degree of 0.1 μmol/L MeJA‐treated fruits was about the same as CK. In contrast, 1 and 10 μmol/L MeJA‐treated fruits were lower than other treatments from the intermediate storage stage, but the effect of low concentration was slightly better; the CI degree was 40.6% lower than CK at 12 days. Total soluble solids and sugar generally reflect the quality of fruits and vegetables. In CK and MeJA treatments, soluble sugar content decreased during storage time. While 1 μmol/L MeJA treatment maintained a higher soluble sugar content than the CK, 10 μmol/L MeJA treatment presented a higher total soluble solids content.

**TABLE 1:** Effect of different concentrations of MeJA treatment on quality of okra during storage at 4 ± 1°C.

Treatment	Storage time/d	Relative electronic conductivity/%	CI degree	Lignin content/%	Total soluble solids/%	Soluble sugar/OD_540_	Pectin content/%	Chlorophyll content/(mg/g)
CK	0	19.49 ± 1.11^a^	0^a^	22.67 ± 1.453^a^	0.333 ± 0.027^a^	0.04 ± 0.002^a^	1.788 ± 0.069^a^	0.177 ± 0.007^a^
	3	39.11 ± 1.7^a^	0.058 ± 0.008^ab^	20.43 ± 4.472^ab^	0.367 ± 0.027^a^	0.113 ± 0.002^a^	1.647 ± 0.012^a^	0.182 ± 0.02^c^
	6	48.00 ± 0.57^a^	0.225 ± 0.014^a^	20.8 ± 4.472^a^	0.367 ± 0.027^a^	0.047 ± 0.006^a^	1.315 ± 0.034^b^	0.171 ± 0.009^a^
	9	51.24 ± 0.66^a^	0.333 ± 0.017^b^	24.83 ± 1.453^a^	0.333 ± 0.027^a^	0.023 ± 0.001^a^	1.346 ± 0.022^b^	0.166 ± 0.003^a^
	12	55.14 ± 0.62^a^	0.492 ± 0.008^a^	21.8 ± 1.268^a^	0.133 ± 0.021^a^	0.03 ± 0^a^	1.033 ± 0.002^c^	0.159 ± 0.002^a^
0.1 μmol/L MeJA	0	19.49 ± 1.11^a^	0^a^	22.67 ± 1.453^a^	0.333 ± 0.027^b^	0.04 ± 0.002^a^	1.788 ± 0.069^a^	0.177 ± 0.007^a^
	3	36.58 ± 0.5^ab^	0.092 ± 0.12^a^	21.53 ± 1.110^ab^	0.4 ± 0^a^	0.147 ± 0.002^a^	1.8 ± 0.153^a^	0.185 ± 0.003^bc^
	6	46.95 ± 0.68^a^	0.233 ± 0.017^a^	22.83 ± 1.106^a^	0.4 ± 0^a^	0.063 ± 0.011^a^	1.72 ± 0.132^ab^	0.177 ± 0.023^a^
	9	51.59 ± 0.68^a^	0.442 ± 0.017^a^	20.47 ± 2.006^a^	0.367 ± 0.022^a^	0.025 ± 0.011^ab^	1.445 ± 0.005^b^	0.171 ± 0.014^a^
	12	55.55 ± 1.06^a^	0.483 ± 0.008^a^	20.53 ± 0.608^a^	0.333 ± 0.024^a^	0.03 ± 0.001^a^	1.19 ± 0.018^b^	0.159 ± 0.009^a^
1 μmol/L MeJA	0	19.49 ± 1.11^a^	0^a^	22.67 ± 1.453^a^	0.333 ± 0.027^a^	0.04 ± 0.002^a^	1.788 ± 0.069^a^	0.177 ± 0.007^a^
	3	35.28 ± 1.7^ab^	0.05 ± 0.014^ab^	23.53 ± 0.242^a^	0.433 ± 0.027^a^	0.154 ± 0.002^b^	1.954 ± 0.169^a^	0.202 ± 0.009^b^
	6	46.59 ± 0.04^a^	0.117 ± 0.022^b^	21.07 ± 0.731^a^	0.433 ± 0.027^a^	0.071 ± 0.007^a^	1.865 ± 0.169^a^	0.181 ± 0.003^a^
	9	50.00 ± 0.32^a^	0.258 ± 0.008^c^	19.87 ± 1.816^a^	0.367 ± 0.016^a^	0.034 ± 0.002^b^	1.649 ± 0.01^a^	0.174 ± 0.006^a^
	12	51.52 ± 0.5^b^	0.292 ± 0.008^c^	18.8 ± 2.074^b^	0.333 ± 0.025^a^	0.04 ± 0^a^	1.493 ± 0.006^a^	0.172 ± 0.001^a^
10 μmol/L MeJA	0	19.49 ± 1.11^a^	0^a^	22.67 ± 1.453^a^	0.333 ± 0.027^a^	0.04 ± 0.002^a^	1.788 ± 0.069^a^	0.177 ± 0.007^a^
	3	31.67 ± 1.8^b^	0.033 ± 0.008^b^	19.2 ± 0.685^c^	0.467 ± 0.022^a^	0.123 ± 0.005^b^	1.716 ± 0.188^a^	0.219 ± 0.003^a^
	6	42.27 ± 0.77^b^	0.158 ± 0.008^b^	20.17 ± 2.103^a^	0.467 ± 0.028^a^	0.049 ± 0.002^a^	1.468 ± 0.055^ab^	0.193 ± 0.003^a^
	9	45.16 ± 1.08^b^	0.275 ± 0.144^c^	21.67 ± 1.110^a^	0.433 ± 0.027^a^	0.028 ± 0.001^b^	1.414 ± 0.01^b^	0.171 ± 0.008^a^
	12	47.13 ± 0.98^c^	0.433 ± 0.008^b^	18.73 ± 2.437^a^	0.233 ± 0.027^ab^	0.03 ± 0^a^	1.118 ± 0.051^bc^	0.166 ± 0.003^a^

*Note*: The results represented as average ± SE; different letters denote significant differences between different treatment at the same time (*p* < .05).

It has been observed that low temperature generally decreases the generation of lignin and pectin. With regard to lignin content compared to CK, 0.1 and 1 μmol/L MeJA treatments increased the lignin content during the early storage period, then decreased. While 10 μmol/L MeJA maintained the lower level. As for pectin content, the changes of 0.1 and 1 μmol/L MeJA treatments showed a similar trend with lignin content, but they were higher than CK. In terms of chlorophyll content, it increased to various degrees in each treatment at 3 days and then decreased, whereas MeJA slowed the decline of chlorophyll content. Treatments with 1 and 10 μmol/L showed significant differences (*p* < .05).

### 3.2 KMO and Bartlett's test

The value transformed by the subordinative function was tested for the correlation between variables by KMO and Bartlett's test. As shown in Table 2, the KMO was 0.758, suggesting that the value was adequate for factor analysis. Furthermore, Bartlett's test had a statistical value of 0.000 and <0.01, indicating that the data were correlated and could be utilized for factor analysis.

**TABLE 2:** KMO and Bartlett's test.

KMO measure of sampling adequacy	Bartlett's test of sphericity		
	Approximate *x* ^2^	*Df*	Sig.
0.758	82.108	21	0.000

In this research, PCA was performed after standardizing quality indicators of different treatments in okra during storage time. The eigenvalues and variance contribution rate are displayed in Table 3. It can be seen from Table 3 that the eigenvalues and cumulative of the first and second components were 4.574, 65.344%, and 1.068, 15.251%, respectively. The cumulative contribution rate was 80.595%, manifesting that the two common factors could be used to replace the whole seven quality indicators better to evaluate fruit quality.

**TABLE 3:** The eigenvalue and variance contribution rate by factor analysis.

Component	Initial eigenvalues			Extraction sums of squared loadings			Rotation sums of squared loadings		
	Total	% of variance	Cumulative	Total	% of variance	Cumulative	Total	% of variance	Cumulative
1	4.574	65.344	65.344	4.574	65.344	65.344	4.534	64.774	64.774
2	1.068	15.251	80.595	1.068	15.251	80.595	1.107	15.821	80.595
3	0.584	8.348	88.943						
4	0.358	5.115	94.057						
5	0.248	3.546	97.603						
6	0.106	1.509	99.112						
7	0.062	0.888	100						

To thoroughly examine the reciprocity between multiple quality indices of okra under varying MeJA concentrations, the regression coefficient approach was employed to construct the component coefficient matrix. In Table 4, the first principal component was mainly positively affected by pectin, soluble sugar, total solids, and chlorophyll and negatively influenced by relative electric conductivity and browning degree. This revealed that the higher levels of pectin, soluble sugar, total soluble solids, and chlorophyll, the better quality of the fruit of the corresponding treatment. However, the higher the relative electric conductivity, the worse the quality. The factor score model was obtained:

(1) $$F_{1}=-0.167X_{1}+0.187X_{2}+0.19\;X_{3}+0.185\;X_{4}+0.219\;X_{5}-0.061\;X_{6}-0.205X_{7}$$

(2) $$F_{2}=-0.096X_{1}+0.062\;X_{2}+0.135\;X_{3}+0.123\;X_{4}+0.248\;X_{5}-0.905\;X_{6}-0.41X_{7}$$

With the variance contribution rate of the two principal component factors as the weight number, the comprehensive score was calculated following formula (3):

(3) $$F=\frac{F_{1}\times \sqrt{4.574}\times 65.344\%+F_{2}\times \sqrt{1.068}\times 15.251\%}{80.595\%}$$

**FIGURE 1:**
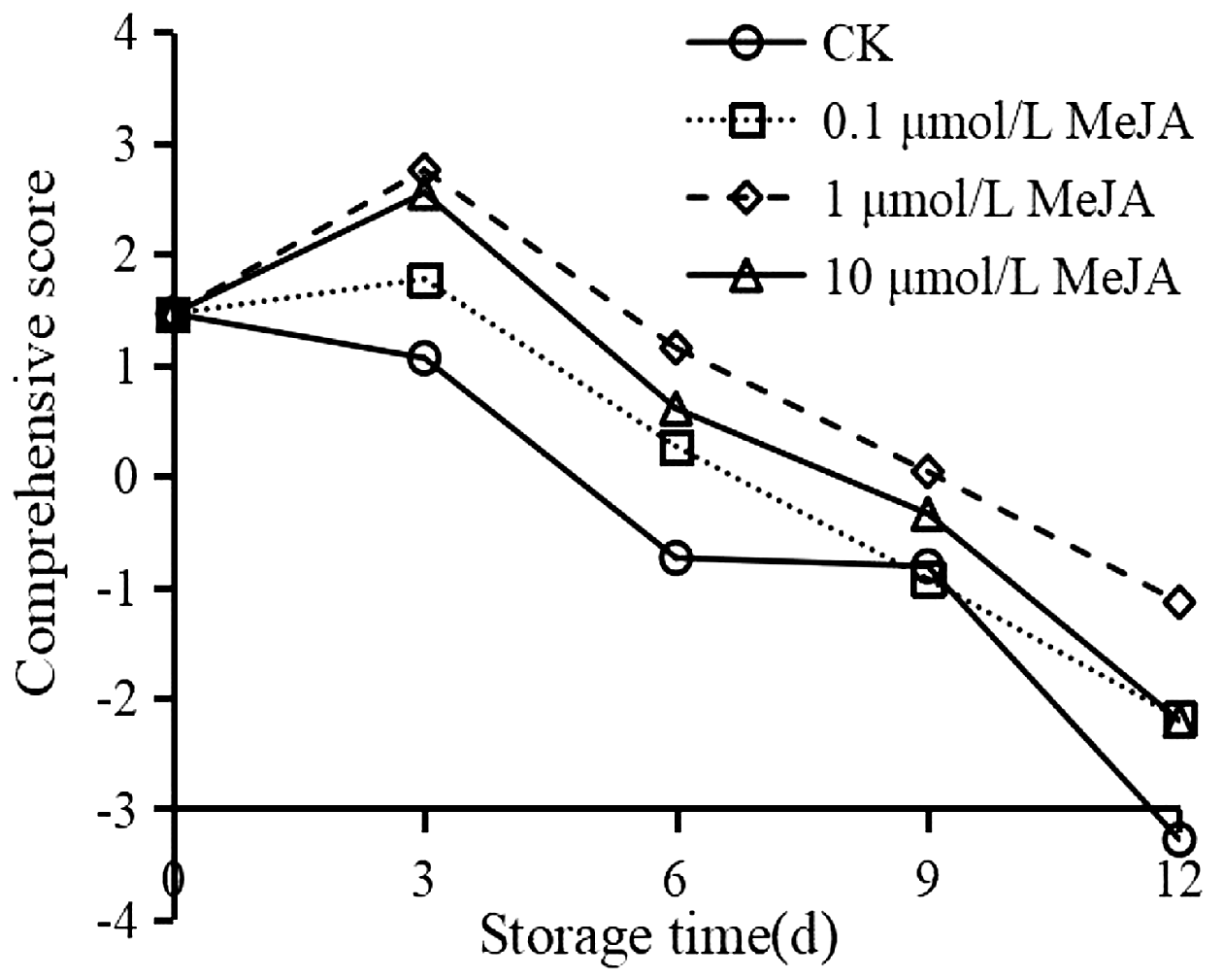
Comprehensive score of different treatments on quality properties in cold‐stored okra.

The comprehensive score and ranking are shown in Table 5 and Figure 1. As Figure 1 shows, the quality in the CK group declined with the extension of storage time, while MeJA treatments retarded the downward trend. Moreover, the comprehensive score of CK appeared as a negative value after 6 days; the negative value appeared until 9 days in MeJA treatments (Table 5), and 1 μmol/L MeJA represented the highest score during the whole storage time. On this basis, fruits with 1 μmol/L MeJA treatment maintained the best quality during cold storage. 1 μmol/L MeJA was screened for the subsequent analysis.

**TABLE 4:** Coefficient matrix of component score.

	Component	
	1	2
Zscore (relative electric conductivity) (*X*_1_)	−0.167	−0.096
Zscore (soluble sugar) (*X*_2_)	0.187	0.062
Zscore (total soluble solid) (*X*_3_)	0.19	−0.135
Zscore (pectin) (*X*_4_)	0.185	0.123
Zscore (chlorophyll) (*X*_5_)	0.219	−0.248
Zscore (lignine) (*X*_6_)	−0.061	0.905
Zscore (CI) (*X*_7_)	−0.205	−0.041

**TABLE 5:** The comprehensive score and ranking of different treatments during storage.

Concentration of MeJA (μmol/L)	Storage time (days)	Comprehensive score	Rank
	0	1.527323429	4
0	3	1.184706276	5
	6	−0.564796522	11
	9	−1.090613053	13
	12	−3.03126584	17
0.1	3	1.843831611	3
	6	0.399572464	8
	9	−1.170927416	14
	12	−2.476235647	16
1	3	2.77910839	1
	6	1.047928368	6
	9	−0.347618385	10
	12	−1.075927328	12
10	3	2.737534015	2
	6	0.783964197	7
	9	−0.328360386	9
	12	−2.218243469	15

### 3.3 Effect of MeJA on total phenolic, flavonoid content in okra

**FIGURE 2:**
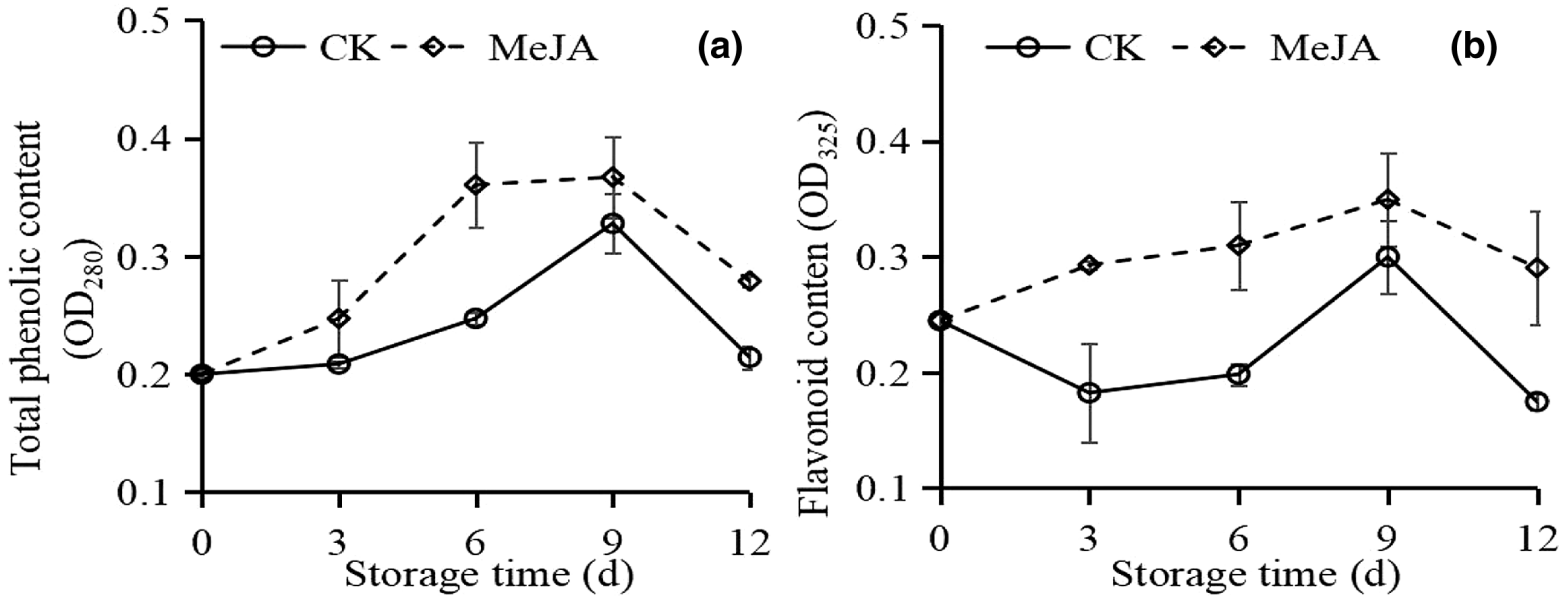
Effect of MeJA on total phenolic content (a) and flavonoid content (b) in okra.

The total phenolic (Figure 2a) and flavonoid content (Figure 2b) of CK and MeJA‐treated fruit rose until 6 or 9 days, then dropped at the end of the storage. In contrast to CK, MeJA‐treated fruit increased throughout the storage time.

### 3.4 Effect of MeJA on MDA and H_2_O_2_ content in okra

**FIGURE 3:**
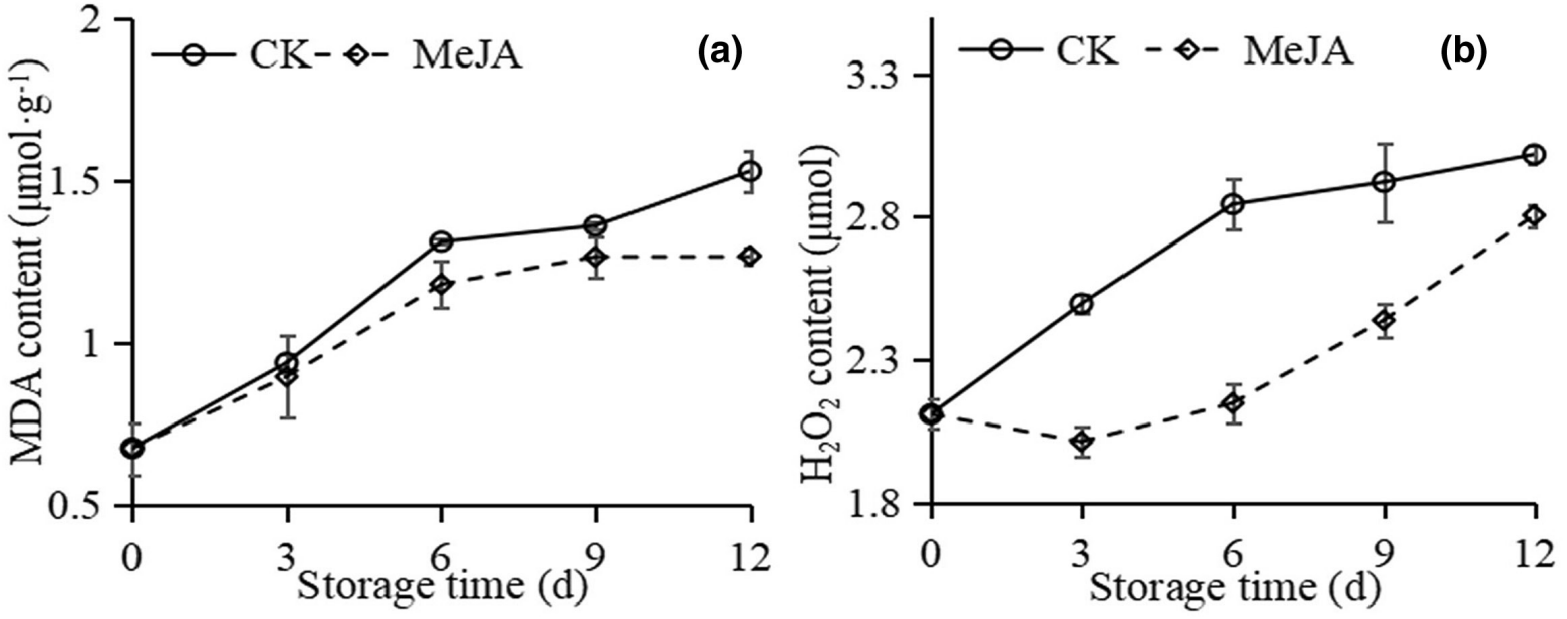
Effect of MeJA on MDA content (a) and H_2_O_2_ content (b) in okra.

For the MDA and H_2_O_2_ content, both CK and MeJA‐treated okra increased (Figure 3), and there was no discernible difference in MDA content between CK and MeJA treatment within 3 days (Figure 3a); however, the H_2_O_2_ content of MeJA‐treated was lower than CK during storage time, dramatically in 3, 6, 9 days (Figure 3b).

### 3.5 Effect of MeJA on SOD, CAT, APX, and POD activity in okra

**FIGURE 4:**
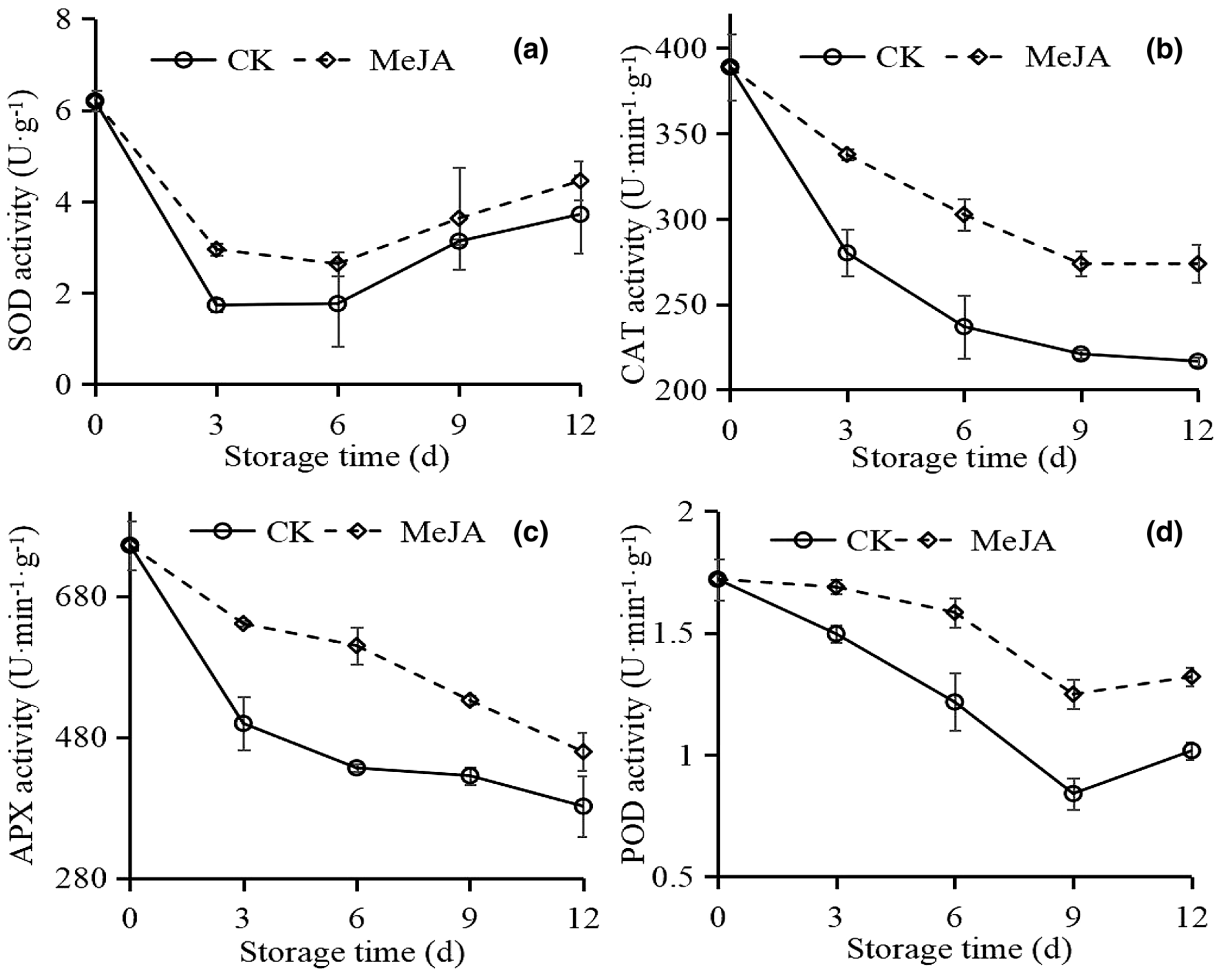
Effect of MeJA on SOD activity (a), CAT activity (b), APX activity (c), and POD activity (d) in okra.

The SOD activity in both control and MeJA‐treated fruit decreased sharply in the first 3 days and then increased until the end of the storage time, whereas MeJA‐treated fruit maintained higher SOD activity throughout the storage period (Figure 4a).

The changes in CAT activity were similar to APX activity (Figure 4b,c). The activity gradually declined during the storage period. In contrast to CK, MeJA‐treated retained a higher level during storage time.

Figure 4d showed that POD activity presented a downward trend in both MeJA and CK groups. Nevertheless, 1 μmol/L MeJA slowed down this trend and was always higher than CK.

### 3.6 Effect of MeJA on PPO activity in okra

**FIGURE 5:**
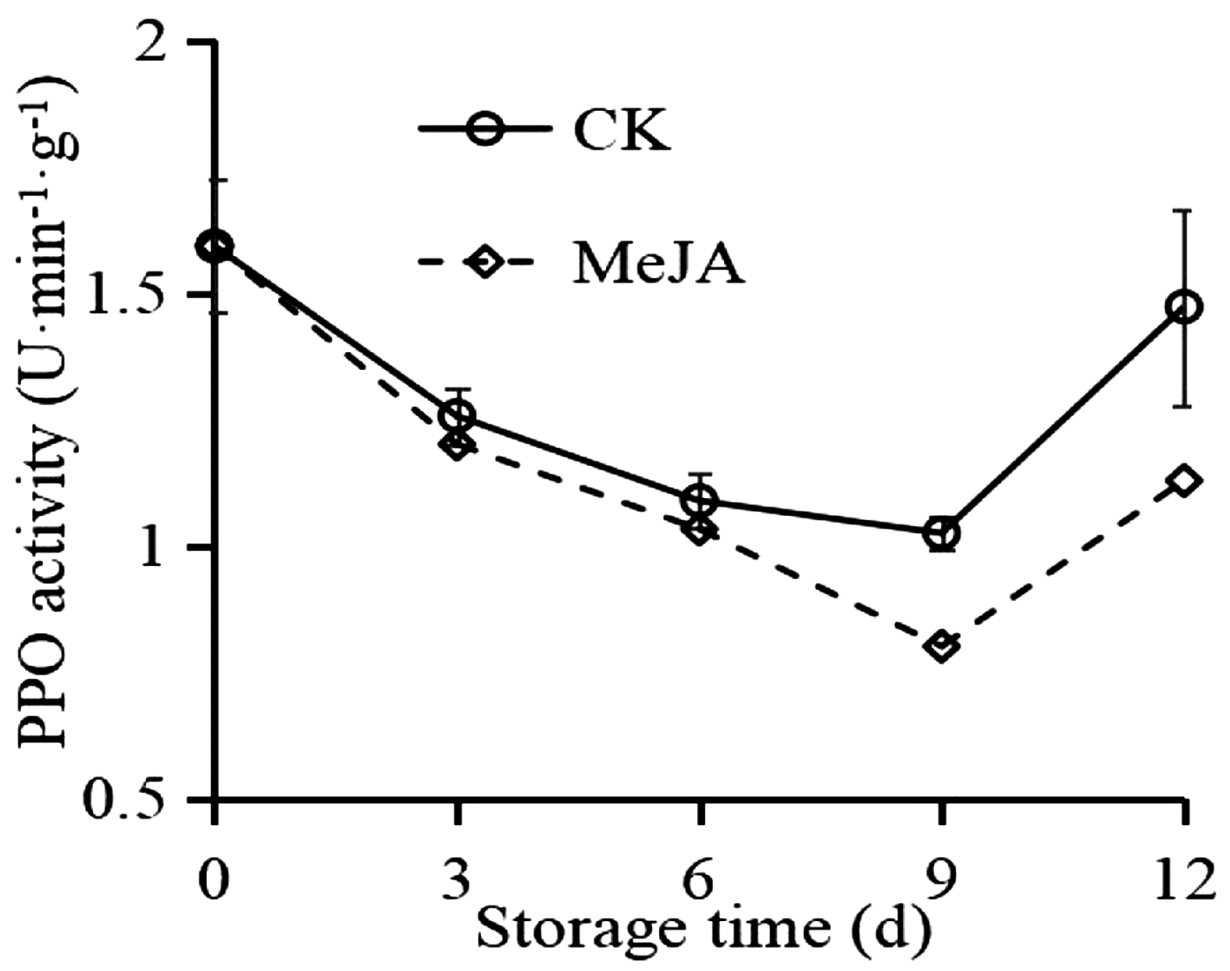
Effect of MeJA on PPO activity in okra.

As observed from Figure 5, PPO activity changed similarly to POD activity, which decreased firstly and then increased. However, compared to CK, the PPO activity of MeJA treatment was lower, especially after 6 days.

### 3.7 Correlation analysis

The correlation of the physiological indexes during the storage of okra showed in Table 6. The storage time displayed an extremely notable positive correlation with MDA content (*p* < .01), a significant positive correlation with H_2_O_2_ content (*p* < .05), and a remarkably negative correlation with CAT, APX, and POD activity (*p* < .05). Total phenolics had a highly significant positive correlation with flavonoid (*p* < .01) and a highly significant negative correlation with PPO activity (*p* < .01). MDA content had a highly significant positive correlation with H_2_O_2_ and a negative correlation with CAT, APX, and POD activity (*p* < .01). H_2_O_2_ content had a highly significant negative correlation with CAT, APX, and POD activity (*p* < .01).

**TABLE 6:** Correlation analysis of the physiological indexes during storage of okra.

	Storage time	Total phenolic	Flavonoid	MDA	H_2_O_2_	SOD	CAT	APX	POD	PPO
Storage time	1									
Total phenolic	0.388	1								
Flavonoid	0.134	0.829**	1							
MDA	0.908**	0.388	−0.025	1						
H_2_O_2_	0.755*	−0.047	−0.378	0.814**	1					
SOD	−0.045	−0.159	0.217	−0.314	−0.187	1				
CAT	−0.772*	−0.223	0.225	−0.92**	−0.894**	0.515	1			
APX	−0.789*	−0.08	0.315	−0.876**	−0.934**	0.466	0.974**	1		
POD	−0.76*	−0.251	0.08	−0.859**	−0.899**	0.197	0.91**	0.866**	1	
PPO	−0.361	−0.862**	−0.66	−0.403	−0.077	0.477	0.372	0.277	0.293	1

*Note*: ** and * indicate significance at .01 and .05 level, respectively.

These results showed that with the extension of cold storage time, fruits accumulated excessive ROS, phenolics, and cell membrane lipid peroxidation, leading to quality deterioration; the antioxidant defense system was triggered in fruits and vegetables to adjust to cold stress, excessive ROS was removed and maintained the fruit quality.

## 4 DISCUSSION

Usually, fruits and vegetables are stored at low temperatures to extend shelf‐life and preserve their quality, but they easily suffer chilling damage, especially the fruits and vegetables originating from tropical and subtropical regions such as okra pod. Okra pod is easily perishable when stored at ambient temperature, while stored below 10°C undergo physiological disorders and chilling injuries such as discolor, shrinking, and decay (Finger et al., 2008). In this study, the CI degree and REC increased until cold‐stored for 6 days resulting in browning spots and decay (when stored at recovery ambient temperature). In contrast, MeJA treatments repressed the CI development to various extents, especially 1 μmol/L, which markedly decreased the CI degree and REC (Table 1). These results indicated that MeJA could alleviate the injury of the cell membrane from cold stress. Similar results have been demonstrated in MeJA treatment in suppression of CI on cucumber (Liu et al., 2016) and cowpea (Fan, Shi, et al., 2016; Fan, Wang, et al., 2016). In addition, plants relieve chilling injury under low temperature by relying on some osmotica, including TSS, soluble sugar (Zhang et al., 2022). In this study, along with the storage period, the TSS and soluble sugar content increased and decreased, while MeJA treatment depicted a higher content than CK. These findings also coincide with lemon fruit (Liao et al., 2022), which might be due to the carbohydrate metabolism. Another important effect of cold stress on fruit is the abnormal cell wall metabolism, where the pectin fractions are located, which finally leads to the change in fruit texture. Many reports showed that inapplicability storage led to pectin degradation and lignification (Chen et al., 2022; Sun et al., 2021). Our results indicated that the pectin content decreased during the cold storage, whereas MeJA hindered the decreasing progress. Moreover, we found that MeJA treatment limited the lignification of okra pod in our present study (Table 1), which is in accordance with MeJA on loquat (Zhang et al., 2022). The fruits and vegetables always discolor during stored periods, and the color fade was associated with chlorophyll degradation. Previous research showed that the chlorophyll content of cowpea gradually decreased during cold storage, while MeJA could attenuate chlorophyll degradation (Fan, Shi, et al., 2016; Fan, Wang, et al., 2016). Interestingly, the chlorophyll content increased during the initial storage period in our present study, which was inconsistent with many other researches, which might be related to the fact that the okra pods were placed in a light incubator. There was a report indicating that the postharvest fruits and vegetables enhanced the chlorophyll content, tissue integrity, and green color at light/dark cycles stimuli (Liu et al., 2015).

As comprehensive evaluation is widely used in fruit and vegetable quality (Lei et al., 2018; Xue et al., 2020), in addition, the factor analysis makes the concept of dimensionality reduction by substituting a limited number of extracted factors for the majority of the original indicators in order to thoroughly assess the fruit quality. According to Table 1, MeJA treatments could effectively mitigate the chilling injury and maintain the storage quality. Subsequently, we applied the comprehensive evaluation to estimate which concentration works best. Table 5 and Figure 1 showed that a comparison with the comprehensive score after different MeJA concentrations found that 1 μmol/L MeJA worked best. Furthermore, similar results were found in MeJA‐treated pineapple (Sangprayoon et al., 2019), strawberry (El‐Mogy et al., 2019), and loquat (Jin et al., 2014).

Low‐temperature stress breaks the ROS homeostasis and accumulates a large amount of ROS, which causes lipid peroxidation of the membrane and membrane deterioration (Lin et al., 2021; Naing & Kim, 2021). Furthermore, the membrane permeable increases. Therefore, MDA and H_2_O_2_ content could directly reflect the degree of cold stress. In this study, the okra pod immersed in 1 μmol/L MeJA retarded the accumulation of the MDA and H_2_O_2_ content (Figure 2), as well as slowed down the increase of relative electric conductivity (Table 1). These results agreed with the previous research of MeJA‐treated pitaya (Li et al., 2018), pineapple (Sangprayoon et al., 2019). These results suggested that MeJA could maintain the cell membrane integrity in fruits at low temperature.

Plants activate their antioxidant defense system to withstand the damage caused by excessive ROS accumulated at low temperatures. The antioxidant defense system consists of antioxidant enzymes and antioxidant substances. The antioxidant enzymes, including SOD, CAT, and APX, play an essential role in scavenging ROS. The superoxide anion was converted to H_2_O_2_ by SOD in the plant, decomposing by CAT and APX (Singh & Singh, 2013). Correlation analysis results indicated that MDA and H_2_O_2_ content significantly negatively correlated with CAT, APX, and POD activity (Table 6). Some researchers have found that NO (Sun et al., 2021), putrescine (Phornvillay et al., 2019), and 1‐MCP (Huang et al., 2012) can reduce ROS accumulation by increasing the SOD, CAT, APX, and POD activity in okra, thus maintaining cell integrity at low temperatures. Moreover, in this study, the higher SOD, CAT, APX, and POD activity was found in 1 μmol/L MeJA‐treated okra (Figure 4). Similarly, MeJA can be used as antioxidant induction machinery to reduce chilling damage in loquat (Cao et al., 2010) and cucumber (Liu et al., 2016).

Besides, plants rely on phenol and flavonoid as antioxidants (Banerjee et al., 2005; Kim et al., 2003). In cold‐stored sweet cherries, the MeJA‐treated fruits maintained higher phenolic compounds and flavonoid content (Faizy et al., 2021). Likewise, in the present study, 1 μmol/L MeJA increased the phenol and flavonoid content during the early period of storage (Figure 3). These results might be due to activating the phenylpropane metabolic pathway, promoting phenolic and flavonoid generation (Huang et al., 2015; Wang et al., 2009). In contrast, the declining trend at the end of the storage time may be due to the oxidation of phenolics by PPO and POD. A similar result was seen in MeJA‐treated blueberries (Huang et al., 2015).

During cold storage, the surface of fruits turns brown frequently. It might be due to the oxidation of phenols by PPO, leading to melanin accumulation (Phornvillay et al., 2019). A report showed that in the presence of H_2_O_2_, POD could coordinate with PPO to rapidly oxidize phenols and cause the browning of fruits and vegetables (Phornvillay et al., 2020). As a consequence, inhibiting PPO activity while increasing POD activity could effectively suppress browning (Fan, Shi, et al., 2016; Fan, Wang, et al., 2016; Nguyen et al., 2003). Meanwhile, 1 μmol/L MeJA treatment inhibited the PPO activity (Figure 5), causing a reduction in the consumption of phenols (Figure 2a), which is also why the phenol content is higher in 1 μmol/L MeJA‐treated fruits.

**FIGURE 6:**
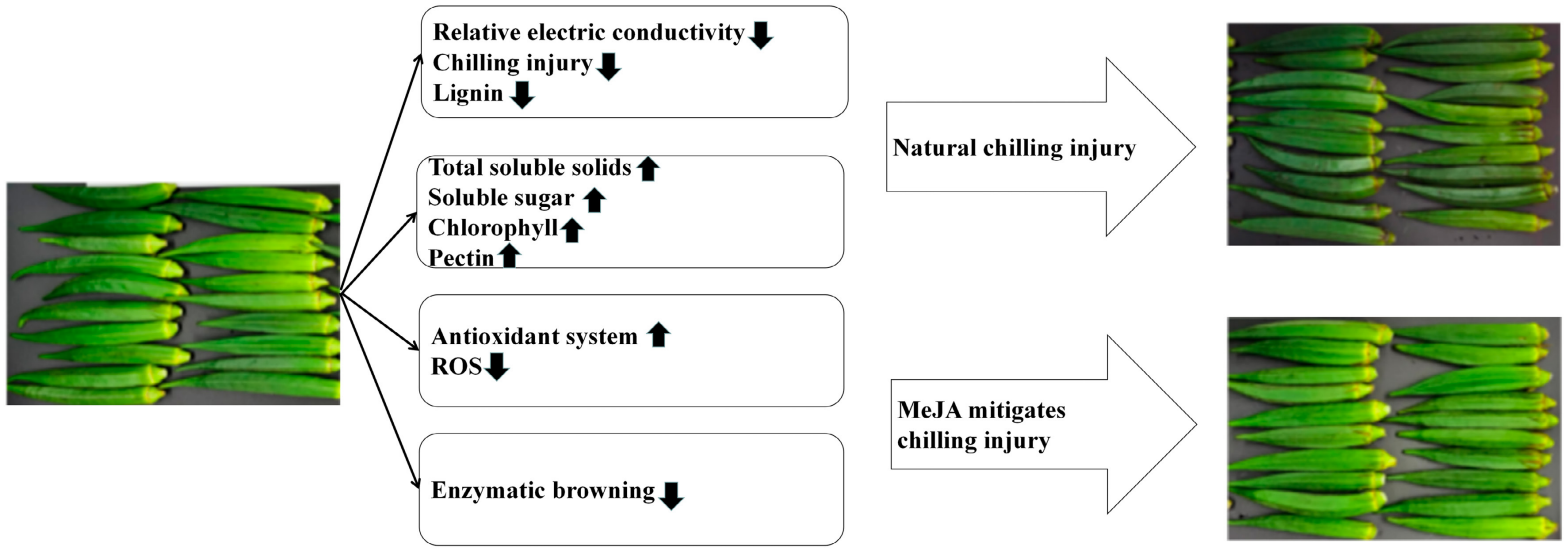
A hypothesized model for regulating mechanism of MeJA treatment in mitigating chilling injury on the okra pod.

Based on the above results, a hypothesized model for regulating mechanism of MeJA treatment in mitigating chilling injury on the okra pod was developed (Figure 6). MeJA treatment reduced relative electric conductivity, chilling injury degree, lignin content, maintained higher TSS, soluble sugar, chlorophyll, and pectin content, activated antioxidant defense system, eliminated excessive ROS, restrained enzymatic browning, and finally attenuated chilling injury of okra pod.

## 5 CONCLUSION

This study evaluated the effects of different concentrations of MeJA on cold‐stored okra fruit. The results indicated that compared with control, MeJA treatment could slow down the increase in REC and CI degree, maintain high TSS, soluble sugar, and pectin content, reduce the degradation of chlorophyll, and inhibit the production of lignin, preserve a better quality. It can be seen from the comprehensive score by factor analysis that the 1 μmol/L MeJA function the best effect, and the REC, CI degree, and lignin content was 6.5%, 40%, and 13.7% of the CK after cold storage for 12 days, respectively.

The correlated physiological indicators demonstrated that MeJA decreased the quality deterioration of okra caused by chilling injury by promoting the production of antioxidants such as total phenolics and flavonoids, inhibiting PPO activity, increasing SOD, CAT, APX, and POD activity, reducing the accumulation of MDA and H_2_O_2_. It is speculated that MeJA could enhance the antioxidant system and modulate the (ROS) homeostasis, thereby attenuating chilling injury and preserving the quality of okra during cold storage.

## CONFLICT OF INTEREST STATEMENT

The authors declare no conflict of interest.

## Data Availability

The data that support the findings of this study are available from the corresponding author upon reasonable request.
